# MNase Profiling of Promoter Chromatin in *Salmonella typhimurium*-Stimulated GM12878 Cells Reveals Dynamic and Response-Specific Nucleosome Architecture

**DOI:** 10.1534/g3.120.401266

**Published:** 2020-05-13

**Authors:** Lauren Cole, Jonathan Dennis

**Affiliations:** Department of Biological Science, Florida State University, Tallahassee, FL 32306

**Keywords:** chromatin, nucleosome, micrococcal nuclease, GM12878, *Salmonella typhimurium*

## Abstract

The nucleosome is the primary unit of chromatin structure and commonly imputed as a regulator of nuclear events, although the exact mechanisms remain unclear. Recent studies have shown that certain nucleosomes can have different sensitivities to micrococcal nuclease (MNase) digestion, resulting in the release of populations of nucleosomes dependent on the concentration of MNase. Mapping MNase sensitivity of nucleosomes at transcription start sites genome-wide reveals an important functional nucleosome organization that correlates with gene expression levels and transcription factor binding. In order to understand nucleosome distribution and sensitivity dynamics during a robust genome response, we mapped nucleosome position and sensitivity using multiple concentrations of MNase. We used the innate immune response as a model system to understand chromatin-mediated regulation. Herein we demonstrate that stimulation of a human lymphoblastoid cell line (GM12878) with heat-killed *Salmonella typhimurium* (HKST) results in changes in nucleosome sensitivity to MNase. We show that the HKST response alters the sensitivity of -1 nucleosomes at highly expressed promoters. Finally, we correlate the increased sensitivity with response-specific transcription factor binding. These results indicate that nucleosome sensitivity dynamics reflect the cellular response to HKST and pave the way for further studies that will deepen our understanding of the specificity of genome response.

The functional role of chromatin is inseparable from a cellular response to a stimulus. The fundamental subunit of chromatin is the nucleosome, composed of a histone octamer core and approximately 150bp of DNA (Kornberg and Lorch 1999). It is commonly asserted that the positional occupancy of nucleosomes can provide access to the underlying DNA sequences, thus affecting nuclear processes (Kaplan *et al.*, 2009). The distribution of nucleosomes across the genome is controlled by chromatin remodeling complexes and DNA sequence (Gupta *et al.*, 2008). However, characterization of the functional organization of the genome remains a major challenge in biology today. Micrococcal nuclease (MNase) was first used to isolate nucleosomal DNA from the chicken beta-globin gene (Sun *et al.*, 1986). It remains the predominant method for generation of nucleosome occupancy maps in eukaryotic genomes, and previous studies have mapped changes in chromatin structure during differentiation, environmental stimulus or stress, and disease states (Shivaswamy *et al.*, 2008; Teif *et al.*, 2012; Druliner *et al.*, 2013; Sexton *et al.*, 2014; Sexton *et al.*, 2016). Additionally, it has been recently shown that nucleosomes exhibit differential sensitivity to MNase. The sensitivity of promoter nucleosomes, particularly the +1 and -1 nucleosomes relative to transcription start- and regulatory factor- binding sites, is a defining chromatin characteristic that gives insight into chromatin-mediated regulation of these loci. The importance of characterizing this differential sensitivity of chromatin has been demonstrated in multiple organisms and is an important variable to consider due to its correlation with transcriptional activity (Vera *et al.*, 2014; Mieczkowski *et al.*, 2016; Chereji *et al.*, 2019; Schwartz *et al.*, 2019).

In order to investigate the role of nucleosome dynamics during the innate immune response, we have mapped nucleosome position and sensitivity at all human promoters in GM12878 lymphoblastoid cells stimulated with heat killed *Salmonella typhimurium* (HKST). We mapped nucleosome distributions with two MNase digestion levels, heavy and light. The comparison of these different MNase digestion levels reveals important information about transcription factor binding, gene expression prediction, and sensitivity of chromatin to digestion (Vera *et al.*, 2014; Mieczkowski *et al.*, 2016; Pass *et al.*, 2017; Brahma and Henikoff 2019). The complicated interplay between chromatin remodeling complexes and the specific epigenetic landscape is largely unknown but likely a major factor in controlling the genetics underlying the diverse kinetics of the immune response (Ramirez-Carrozzi *et al.*, 2006; Natoli 2010; Dorrington and Fraser 2019). Here we show that stimulation of a human lymphoblastoid cell line with HKST causes changes in sensitivity to MNase at specific regulatory nucleosomes flanking the transcription start site (TSS). We find that in the 20 min post-HKST time point the -1 nucleosome becomes significantly more sensitive to MNase in highly expressed genes, and that active RNA Pol II (Pol2s2) as well transcription factor binding at the TSS (NF**κ**B, Pu1, and Ebf1) is associated with flanking nucleosomes sensitive to MNase digestion.

## Materials and Methods

### Cell culture

Cell GM12878 cells were grown at 37° in 15% FBS-supplemented RPMI medium. Cells were stimulated with 1.0 X 10^9^ heat-killed *Salmonella typhimurium* (HKST; 15 min at 80°) for 20 min, 40 min and 60 min and harvested at the end of each time point, in biological replicate (Fig. S1 & S2).

### Cell harvest and nuclei purification

Approximately 1 X 10^7^ cells were harvested, cross-linked in 1% formaldehyde, and incubated for 10 min at room temperature. After the 10 min incubation, the cross-linking reaction was quenched with 125 mM glycine. Next, the nuclei were isolated in nucleus isolation buffer containing: 10 mM HEPES at pH 7.8, 2 mM MgOAc_2_, 0.3 M sucrose, 1 mM CaCl_2_, and 1% Nonidet P-40. The nuclei were then pelleted by centrifugation at 1000g for 5 min at 4°.

### MNase digestion of chromatin

At each time point and biological replicate ∼2.5 X 10^6^ nuclei were treated with light (20U MNase, Worthington Biochemical) and heavy MNase-digestion conditions (200U MNase, Worthington Biochemical), see average fragment size distribution in Fig. S1B. Chromatin at each time point was digested separately with the light and heavy concentrations of MNase for five minutes at 37° and stopped with EDTA. Decrosslinked, protease-digested DNA from MNase-digested nuclei was isolated via phenol-chloroform extraction and mononucleosome sized bands resolved with a 2% TBE agarose gel. The ∼150bp mononucleosomal band was excised for each time point and MNase concentration. Additionally, two untreated control samples were harvested and processed as described, and have been referenced here as untreated samples.

### Mononucleosome DNA Library Preparation

MNase sequencing libraries were prepared for each replicate using NEBNext Ultra DNA library Prep Kit for Illumina (NEB #E7370S); using 30ng of input mononucleosomal DNA from each digestion level and time point. Following end-prep and adaptor ligation, the libraries were purified with AMPure XP beads. Universal and index primers from NEBNext Multiplex Oligos for Illumina (NEB #E7335S) were incorporated by a 12 cycle PCR. Library size and quality was verified with the Agilent 2100 Bioanalyzer. Molar concentration of each indexed library was determined by KAPA quantitative PCR and size corrected using sizing information from the Bioanalyzer.

### Solution-based sequence capture and Illumina flowcell hybridization and sequencing

Previously, we combined MNase-seq with in-solution targeted enrichment of 2 kb surrounding TSSs of 21,857 human genes (Druliner *et al.*, 2016; Sexton *et al.*, 2016), as curated by NCBI RefSeq (Pruitt *et al.*, 2009). We termed this approach Transcription Start Site MNase-seq (mTSS-seq). Size selected fragments (∼50-200 bp) were used to prepare Illumina sequencing libraries and subjected to targeted enrichment utilizing the custom-designed Roche Nimblegen SeqCap EZ Library. DNA fragments were captured according to the Roche Nimblegen protocol (https://sequencing.roche.com/en/products-solutions/by-category/target-enrichment/hybridization/seqcap-ez-choice.html). By qPCR we observe ∼300 fold enrichment of sample target genes compared to off-target loci. Paired-end reads (see below) were aligned to the hg19 genome assembly (IHGSC 2001).

### HiSeq 2500 data processing

Illumina adapters were clipped and aligned to the HG19 genome assembly, with unpaired and non-uniquely aligned reads discarded (bowtie2 v2.1.0, samtools v1.3). Mononucleosome-sized fragments were used to infer nucleosome position. Nucleosome occupancy profiles were obtained by calculating the fragments per million that mapped at each base-pair in the SeqCap regions (bedtoolsCoverage). Midpoints for nucleosome distributions were determined through the calculation of center fragments in 60 bp windows at a 10 bp step in the 2kb surrounding each TSS. Data matrices were subsequently processed in R (https://github.com/dvera genmat package, using matOps, matHeatmap). matHeatmap was used to plot normalized rpm mTSS-seq data for all RefSeq genes +/− 2kb surrounding the annotated TSSs. Average plots represent the average value of all promoters in a respective cluster in 10bp windows across the 2kb SeqCap region. Gene ontology analyses based on heatmap gene classifications were performed using two unranked sets of genes, the target set of genes and the total mTSS-seq gene list as background (Eden *et al.* 2009).

### Data availability

The data discussed in this publication have been deposited in NCBI’s Gene Expression Omnibus (Edgar *et al.*, 2002) and are accessible through GEO Series accession number GSE139224. Supplemental material available at figshare: https://doi.org/10.25387/g3.12290333.

## Results

### Total nucleosome occupancy profiles are similar during HKST stimulation of B-lymphoblastoid cells

**Figure 1:**
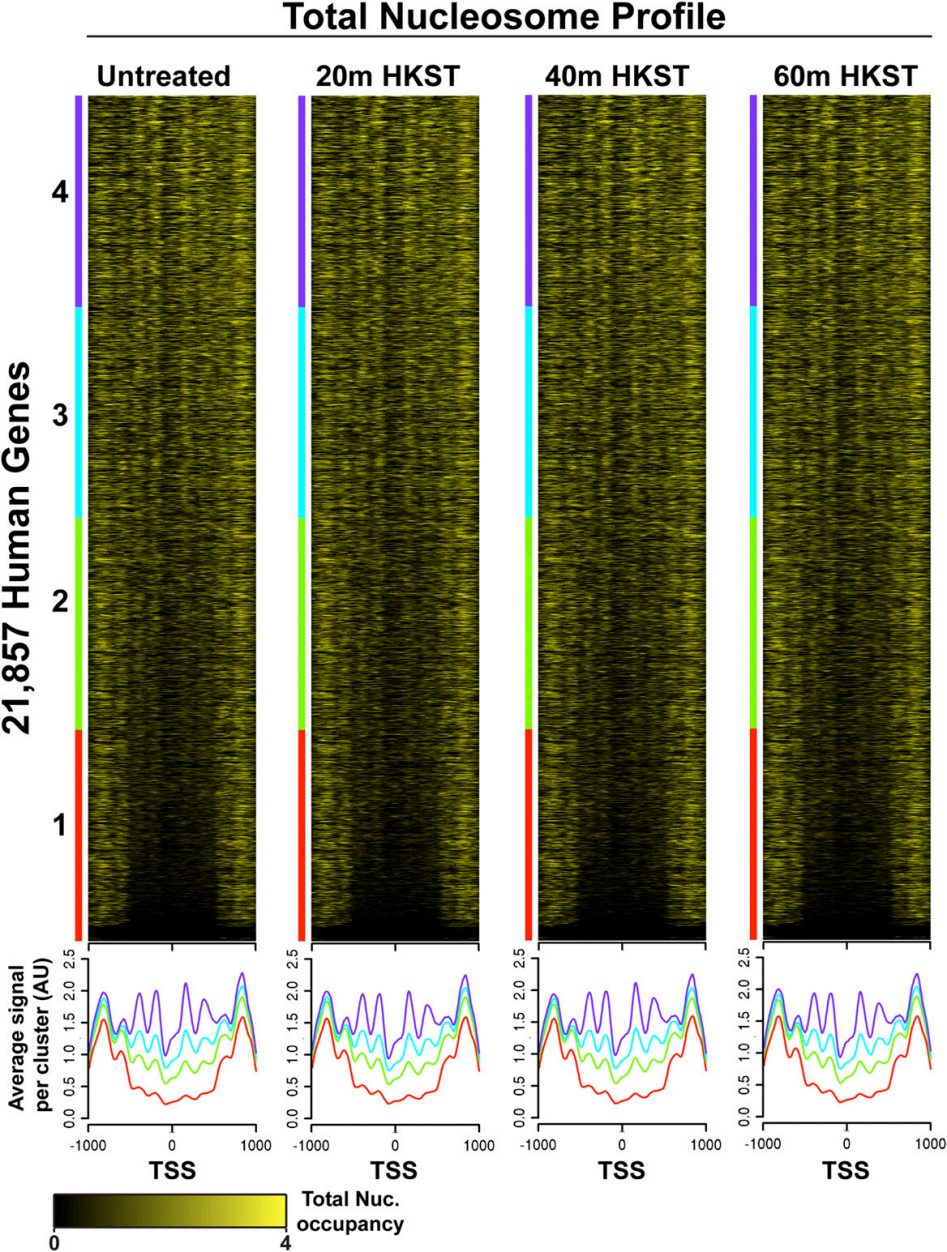
Nucleosome occupancy during time course stimulation with HKST. Total nucleosome fragments sorted into quartiles based on maximum signal. All heatmaps sorted in the same order, -1000bp/+1000bp surrounding the TSS for all RefSeq open reading frames. Yellow indicates presence of nucleosomal fragment.

We have mapped nucleosome distribution during the immune response to HKST. MNase Transcription Start Site-enriched sequencing (mTSS-seq) allows for high quality nucleosome maps at all human promoters (Fig. S1). mTSS-seq data are highly concordant with the preeminent nucleosome maps in lymphoblastoid cell lines (Fig. S1C, (Gaffney *et al.*, 2012)). Here we observe that the average nucleosome profiles remain similar between the untreated control and HKST-treated time points (Figure 1). The mTSS-seq HKST time course data displays canonical promoter structure where nucleosomes flank the TSS and the -3,-2, -1, +1, +2, and +3 nucleosomes are clearly observed (Figure 1). While there are slight changes in abundance of specific nucleosomal fragments during the HKST time course, previous work in our lab has shown that nucleosome sensitivity to MNase is associated with promoter activity in Maize (Vera *et al.*, 2014). We did not observe a correlation between gene expression and differential nucleosome occupancy of the untreated and 20 min post-HKST samples (Fig. S3**)**. Our work in human cells as well as recently published work from the van Essen lab indicates that the proportion of inducible promoters is relatively small in the scope of all ∼21,000 human genes and that chromatin structural changes may occur with or without concurrent changes in gene expression (Oruba *et al.*, 2020, Dennis lab, unpublished). This prompted us to look at nucleosome sensitivity dynamics during the immune response to HKST.

### HKST stimulation of B-lymphoblastoid cells results in changes in MNase sensitivity of promoter nucleosomes

**Figure 2:**
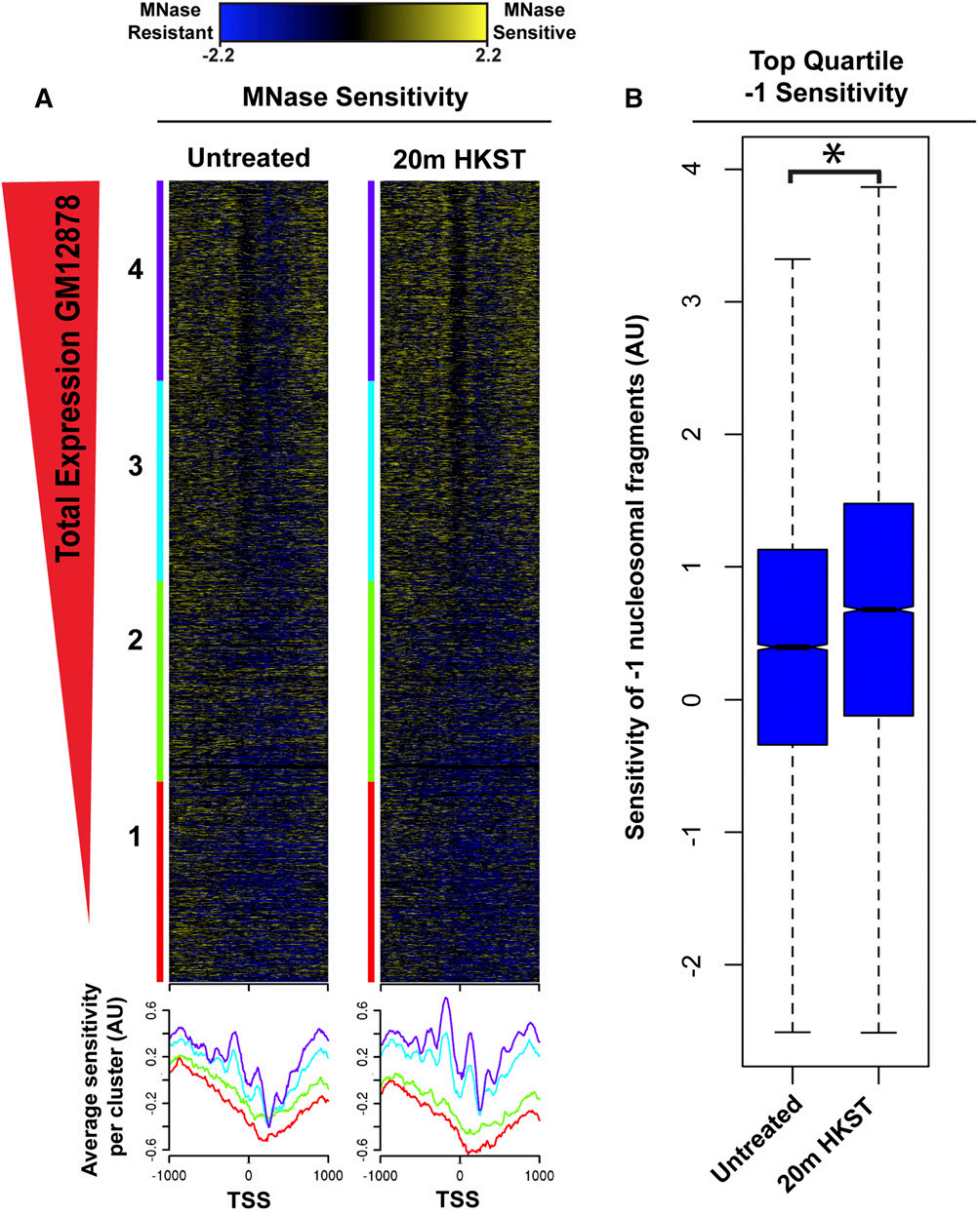
Sensitive nucleosomes are associated with transcription and nucleosome sensitivity changes during HKST stimulation. MNase sensitivity of untreated GM12878 and 20 min HKST-treated cells were sorted into quartiles based on total expression (GSM2344230). Blue indicates MNase-resistant nucleosomal fragments and yellow indicates MNase-sensitive nucleosomal fragments. (B) Boxplot of -1 nucleosome sensitivity data for all promoters in the top quartile of expressed genes from the untreated control and the 20 min post-HKST time point, asterisk represents significance.

We used differences in nuclease sensitivity to distinguish nucleosome-bound fragments that were preferentially released by light digestion (MNase-sensitive fragments, MSFs) from nucleosome-bound fragments that were recovered even after heavy digestion (MNase-resistant fragments, MRFs). To identify MSFs within our mTSS-seq experiment we calculated the log_2_ratio of the light/heavy MNase digests and the resulting positive values correspond to MSFs and the negative values correspond to MRFs. We then sorted the resulting MNase-sensitivity nucleosome profiles based on maximum expression using existing RNAseq datasets for GM12878 cells (Davis *et al.*, 2018). We observed a strongly positioned -1 sensitive nucleosome in the top quartile of maximum transcription and with 20 min HKST treatment, this nucleosome becomes significantly more sensitive to MNase digestion (Figure 2A). We directly compared fragments from -200 to 0 bp relative to the TSS, which represent sensitive -1 nucleosomes, in the 20 min HKST time point and the untreated control in the top quartile of expressed genes. Using a paired *t*-test, we find that the -1 sensitive nucleosomal profiles in the 20 min time point are significantly different from the -1 nucleosomes in the untreated control in these expressed promoters (Figure 2B). In the bottom quartile of low and non-expressed genes we observe promoters are more disorganized and contain less well-positioned nucleosomes (Figure 2A). These disorganized promoters are consistent with recently published work mapping nucleosome organization in *Drosophila* S2 cells (Cheriji *et al.*, 2019). The differences we observe in MNase-sensitivity of well-positioned nucleosomes in promoters of expressed genes at the 20 min HKST time point are consistent with the time frame of a primary response to bacterial infection (Herschman 1991; Winkles 1998). Additionally, it is important to note that independently each time point contains positioned nucleosomes that are either sensitive or resistant to MNase-digestion (Fig. S4). Thus, when the MNase-sensitivity for the untreated control and 20 min post-HKST time points are independently sorted by kmeans clustering based on similar features, we find that they each contain a set of ∼1500 unique promoters that are classified as having a dominant -1 sensitive nucleosome (Fig. S4). Interestingly, we find that the promoters unique to the 20 min post-HKST are ontologically enriched for processes that are specific to transcription initiation and contain immune response genes (Table S1). The promoters unique to the untreated control contain genes that are ontologically enriched for more general processes such as detection of chemical stimulus involved in sensory perception and nucleic acid metabolic process (Table S2) (GOrilla; Eden *et al.*, 2009).

### Sensitivity of TSS-flanking nucleosomes is associated with transcription factor binding

**Figure 3:**
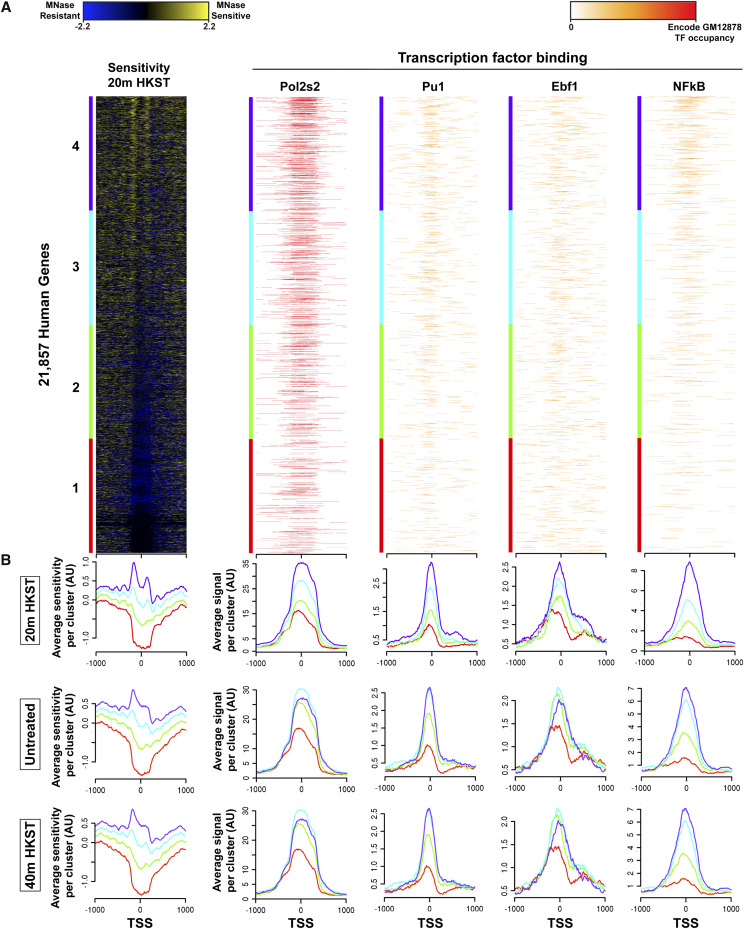
Sensitive nucleosomes flank transcription factor binding sites. (A) MNase-sensitivity of nucleosomes at 20 min post-HKST stimulation was sorted into quartiles based on maximum sensitivity followed by regulatory factor binding for Pol2s2, NFkB, Pu1, and Ebf1 using called narrow peaks from publicly available ENCODE ChIP-seq data on the same sort order. (B) Average plots of MNase-sensitivity of nucleosomes at the untreated and 40 min post-HKST time points followed by average plots of immune transcription factor binding for NFkB, Pu1, Ebf1 in the same sort order.

We next wanted to determine the relationship between MNase-sensitivity and transcription factor binding. As we have associated nucleosome sensitivity with active transcription, we first sorted the 20 min HKST time point into quartiles based on maximum sensitivity surrounding the TSS. We then sorted available regulatory factor data from unstimulated cells for active Pol2, NF**κ**B, Pu1, and Ebf1 in the same gene order (Figure 3) (Davis *et al.*, 2018). Here we show that MSFs flank important immune regulatory factor peaks at the TSS during the immune response to HKST (Figure 3A). The presence of MSFs flanking TF binding sites in the unstimulated state suggests that local chromatin architecture plays a role in regulatory factor binding. We find that the MNase-sensitive flanking nucleosomes in the 20 min post-HKST time point most strongly correspond to the highest occupancy of basal unstimulated binding of Pol2s2, NF**κ**B, Pu1, and Ebf1. However, in the independently sorted untreated control and 40 min post-HSKT samples we do not observe this coupling of TSS-flanking nucleosome sensitivity and regulatory factor occupancy, which further suggests that the chromatin signature at the 20 min post-HKST indicates an early genomic response to HKST (Figure 3B). The presence of sensitive nucleosomes flanking TF binding sites (TFBS) is concordant with similar results from plants and yeast (Zentner and Henikoff 2012; Vera *et al.*, 2014; Pass *et al.*, 2017). These results are consistent with a model in which nucleosomes that provide access to regulatory factor binding sites necessary for a specific genomic response will be more mobile and this will be reflected in greater sensitivity to digestion by MNase.

## Discussion

In this study we measured changes in chromatin structure at promoters in the human lymphoblastoid cell line GM12878 in response to stimulation with HKST. We find that during this time course total nucleosome occupancy across the TSS remains similar (Figure 1). However, we have observed changes in promoter nucleosome sensitivity which occur on a time-scale commensurate with known signaling cascades (Dal Porto *et al.*, 2004; Harwood and Batista 2008; Arpaia *et al.*, 2011; Browne 2012; Bagaev *et al.*, 2019). Specifically, we observed a significant increase of sensitivity of the -1 positioned nucleosome 20 min after stimulation with HKST. Alternations in chromatin structure that result in nucleosome sensitivity to MNase may be the result of transcription machinery, chromatin remodelers, histone chaperones, or other regulatory factors. Our results are consistent with important regulatory functions of -1/+1 nucleosomes reported first in yeast and *Drosophila*, such as maintaining the structure of a nucleosome-free region at the TSS, interaction with RNA Pol II, transcription factor binding and access to underlying DNA (Svaren and Hörz 1995; Jiang and Pugh 2009; Radman-Livaja and Rando 2010; Ballaré *et al.*, 2013; Nie *et al.*, 2014; Voong *et al.*, 2016). In addition to increased sensitivity of these regulatory nucleosomes at 20 min post-HKST, we find an independently sorted set of genes unique to the 20 min time point which have promoters containing distinct sensitive -1 nucleosomes. These unique promoters to the 20 min post-HKST time point are ontologically enriched for RNA Pol II transcription initiation, lysine metabolism, and regulation of T-helper 2 cell differentiation (Fig. S4 & Table S1). It is expected that the immune response to HKST results in the transcription of many genes, and studies have shown essential amino acid metabolism to be critical to proper immune system function (Chen *et al.*, 2003; McGaha *et al.*, 2012; Iseri and Klasing 2014; Han *et al.*, 2018).

MNase-sensitivity changes are likely the direct result of HKST immune stimulus producing an inflammatory signaling cascade that induces chromatin regulatory mechanisms at the appropriate promoters. We also observe that the TSS-flanking sensitive nucleosome occupancy at the 40 and 60 min post-stimulus timepoints begins to return to the untreated chromatin architecture (Fig. S5). These results are consistent with the observation that changes in promoter nucleosome architecture is a transient event (Sexton *et al.*, 2014; Sexton *et al.*, 2016). These results suggest that nucleosome sensitivity may be used as a powerful tool to understand the potential of a cell, beyond the information that is garnered from gene expression.

We have shown that sensitive nucleosomes are associated with active transcription. 20 min after HKST stimulation, we observe significantly greater -1/+1 nucleosome sensitivity that flanks a larger nucleosome-free region at the TSS (Figure 2). These results are consistent with observations of nucleosome structure at active promoters; and, the additional information given by MNase-sensitivity reflects the regulatory potential of loci that contain sensitive nucleosomes, as reported in yeast, plants, and drosophila (Vera *et al.*, 2014; Mieczkowski *et al.*, 2016; Brahma and Henikoff 2019). We demonstrate that immune transcription factors NFkB, Pu1, and Ebf1 are associated with positioned sensitive nucleosomes (Garrett-Sinha *et al.*, 1999; Somasundaram *et al.*, 2015; Willis *et al.*, 2017; Zhang *et al.*, 2017; Schwartz *et al.*, 2019). Time course studies have shown early and late gene expression changes during B-cell activation, commensurate with the chromatin sensitivity changes found at promoters following HKST treatment (Fowler *et al.*, 2015; Hawkins *et al.*, 2013). These changes represent a new biochemical potential for cells, and studies of the misregulation of the IKK/NF**κ**B pathway in lymphomas and leukemias show that signal transduction occurs as quickly as 15 min with TNFα stimulation (Staudt 2010; Hsieh and Van Etten 2014). *In vitro* studies have shown that TF binding to nucleosomal DNA requires nucleosome eviction or repositioning, however pioneer TFs can bind to DNA within the bounds of the nucleosome, which may result in nucleosome sensitivity to MNase (Zaret and Carroll 2011; Iwafuchi-Doi and Zaret 2014; Zhu *et al.*, 2018). NF**κ**B has been studied in depth regarding its ability to bind chromatin and induce immune gene transcription, and it appears to play a diverse role in promoter binding and activation of genes in hetero- and euchromatic regions of the genome (Lone *et al.*, 2013; Bhatt and Ghosh 2014; Cieślik and Bekiranov 2015). The association of these immune TFs with increased sensitive nucleosomes at the 20 min time point suggests that changes in sensitivity reflect a new chromatin landscape at appropriate promoters, potentiating regulatory factor binding.

A genomic response occurs through multiple regulatory layers, including signaling cascades, regulatory factors, and the resulting regulation of chromatin structure and gene expression. Our study adds to a comprehensive understanding of the induction of immune signaling pathways. Our results add important and complementary information to existing data showing the importance of the chromatin landscape at promoters including nucleosome position and sensitivity to MNase, localization of regulatory factors on promoters and histone post-translational modifications. The data we show here expounds upon the interplay between chromatin structure to function, which is consonant with models suggested by preeminent immunologists (Natoli 2010; Cuartero *et al.*, 2018; Zhang and Cao 2019). In aggregate, our results suggest that chromatin structure plays a functional role in the dynamic immune response and that nucleosome sensitivity indicates the regulatory potential of specific loci that are poised for the appropriate genomic response.
